# CRISPR-Cas12a and DNA Tetrahedron Assemblies Amplified Fluorescence Anisotropy for the Sensitive Detection of Hepatitis B Virus DNA

**DOI:** 10.3390/bios15100700

**Published:** 2025-10-17

**Authors:** Yu Qin, Jiali Xie, Shujun Zhen

**Affiliations:** 1 College of Chemistry and Chemical Engineering, Southwest University, Chongqing 400715, China; swuqy1@email.swu.edu.cn (Y.Q.); xji0208@email.swu.edu.cn (J.X.); 2 Key Laboratory of Biomedical Analytics of Chongqing Science and Technology Bureau, Southwest University, Chongqing 400715, China; 3 Academy for Advanced Interdisciplinary Studies, Southwest University, Chongqing 400715, China

**Keywords:** Fluorescence anisotropy, hepatitis B virus, CRISPR, DNA tetrahedron

## Abstract

Fluorescence anisotropy (FA) has been widely used for analyzing biomolecules due to its high throughput, homogeneous detection, and strong resistance to photobleaching. However, the traditional FA method suffers from low sensitivity when the target molecules are small and rotate rapidly, often producing insignificant changes in the FA value. In this study, by combining double signal amplification through the trans-cleavage of CRISPR-Cas12a and DNA tetrahedron assemblies with a large molecular size, a new, fast, simple and highly sensitive FA method was constructed to achieve the quantitative detection of hepatitis B virus DNA (HBV-DNA). The experimental results showed that the linear range of this method was 0.5–9 nmol/L, and the detection limit (LOD = 3*σ*/*k*) was 48 pmol/L. In addition, the method demonstrated excellent selectivity and anti-interference, and it was successfully applied to detect HBV-DNA in human serum, indicating that this method has the potential for clinical diagnosis.

## 1. Introduction

Fluorescence anisotropy (FA), also referred as fluorescence polarization, describes a phenomenon in which fluorescent molecules emit light with varying intensities along different polarization axes [1,2]. The FA assay is a ratiometric technique. Compared with traditional fluorescent methods, it has greater resistance to photobleaching and signal fluctuations. Moreover, unlike the lateral flow chromatography method used for heterogeneous detection, FA is a homogeneous detection method that can be applied by mixing the sample, fluorescently labeled probe, and analyte in a solution for direct measurements, without the need for separation and washing steps. Therefore, it can be directly used to analyze complex samples [3,4,5,6,7,8,9]. Based on these advantages, FA methods have been widely used in protein detection, drug screening, food analysis, environmental monitoring and biochemical research [10,11].

Nucleic acid probes are biorecognition tools constructed from nucleic acid sequences. They have specific recognition capabilities and are constructed as single-stranded DNA (ssDNA) or RNA, as well as aptamers [12,13,14]. Because of the high affinity and specificity of recognition by nucleic acid probes, they are usually modified with fluorescent dyes when used as the recognition unit in an FA method. Biomolecules are detected by the change in the anisotropy (*r*) generated before and after binding to the target [15]. Although nucleic acid probe-based FA methods have become an important means of detecting biomolecules, the small change in *r* of the nucleic acid probe after binding with a target molecule and the high background *r* of the nucleic acid probe itself have led to the low sensitivity of the FA method.

In recent years, clusters of regularly interspaced short palindromic repeats (CRISPR) and CRISPR-associated protein (Cas) systems were discovered in bacteria and archaea and have become widely used in bioanalysis and diagnostics [16,17]. By combining the CRISPR/Cas system with nucleic acid amplification technology or nanomaterials, high-sensitivity detection of specific viral nucleic acid sequences has been achieved, with the signal output generated by fluorescence, lateral flow chromatography test strips, electrochemistry, or colorimetry [18,19,20,21]. Researchers are also committed to combining nucleic acid-free extraction and amplification techniques with CRISPR/Cas systems to further improve the sensitivity of detection [22,23]. Portable and automated detection is also being achieved by simultaneously integrating microfluidic technology into detection systems [24,25]. In addition, research is focused on further enhancing the ability to detect multiple molecules in one reaction system, making it possible to simultaneously detect multiple pathogens [26,27,28]. Cas12a is a typical class 2 effector protein that binds to CRISPR RNA (crRNA) and non-specifically cleaves (trans-cut) ssDNA into small fragments after recognizing the DNA target [29,30,31,32]. As a result, a large number of DNA fragments with a small mass (or volume) are generated. The CRISPR/Cas12a system is unique in that it combines the benefits of a homogeneous reaction and the trans-cleavage activity provided by the Cas12a enzyme [33,34,35]. Therefore, based on these advantages, the CRISPR/Cas12a system can be used as an effective method to reduce the background FA to improve the sensitivity of the FA method, which has been previously confirmed by our group [36,37]. However, the signal generated by the FA method depends on variations in *r*. Because the method can only produce limited amplitude changes based on low background values, this results in a relatively low overall signal-to-background ratio (SBR), rendering it difficult to distinguish weak positive signals from background noise, thereby limiting the method detection limits and sensitivity.

DNA tetrahedrons (TDFs) have a uniform, rigid, and massive structure. Its initial FA background value is both high and extremely stable, with little environmental interference. TDFs can produce statistically significant FA changes, improving detection, significantly reducing the detection limit, and increasing detection sensitivity. Thus, in this study, to further improve the sensitivity of the CRISPR/Cas12a-based FA method, the structure formed by TDFs and a DNA cross scaffold with a large mass (or volume) was used to enhance FA signals. The constructed FA method was applied to the sensitive detection of hepatitis B virus DNA (HBV-DNA). When HBV-DNA was present, the CRISPR/Cas12a system was activated to cleave the ssDNA on the fluorescent group-modified DNA cross scaffold into small fragments, and *r* was reduced significantly. When the target HBV-DNA was absent, the CRISPR/Cas12a system was not activated. Therefore, the DNA cross scaffold maintained its intact structure and remained complexed with TDFs, and thus maintained a greater mass (or volume) that produced a higher *r*. By clearly reducing *r*, HBV-DNA was detected at concentrations of 0.5 nmol/L to 9 nmol/L, with a limit of detection (LOD) as low as 48 pmol/L.

## 2. Materials and Methods

### 2.1. Reagents and Apparatus

PBS buffer (1×; 136.7 mmol/L NaCl, 2.7 mmol/L KCl, 8.72 mmol/L Na_2_HPO_4_, 1.41 mmol/L KH_2_PO_4_), TBE buffer (1×; 89 mmol/L Tris-boric, 2 mmol/L EDTA; pH 8.2), diethyl pyrocarbonate (DEPC) water, acrylamide/methylenedioxybisacrylamide 40% solution (29:1), and 4S Red Plus nucleic acid stain (10,000× aqueous solution) were purchased from Sangon Biotech Company, Ltd. (Shanghai, China). crRNA and DNA oligonucleotides were synthesized and purified by Sangon Biotech Company, Ltd. (Shanghai, China), with the sequences listed in Table S1.

D-Phenylalanine (D-Phe), glutamic acid (Glu), glutathione (GSH), lysine (Lys), and ATP were purchased from Shanghai Aladdin Biochemical Science and Technology Co., Ltd. (Shanghai, China). Bovine serum albumin (BSA) and trypsin were purchased from Sigma Aldrich (Shanghai) Trading Co. Human serum albumin (HSA) was purchased from Beijing Huameike Biotechnology Co. The chemical reagents used in the experiments were of analytical grade, and the water used in the experiments was Milli-Q ultrapure water (18.2 MΩ·cm) from Millipore Systems.

The concentrations of the oligonucleotide solutions were confirmed using a Nanodrop 2000 spectrophotometer (Thermo Fisher, Shanghai, China). The temperature was controlled using a Thermomixer C (Eppendorf, Hamburg, Germany). The solutions were homogeneously mixed using an MX-E mixer (Haimen, Nantong, China). Fluorescence intensity was measured using an F-2500 fluorescence spectrophotometer (Hitachi, Tokyo, Japan) with a polarizing filter. The gels were electrophoresed in a DYCZ-24DN electro-phoresis apparatus (Liuyi Instrument Co., Beijing, China) and photographed on a BG-gdsAUTO520 imager (Baygene Biotechnologies Co., Shanghai, China).

### 2.2. FA Analysis

The fluorescence intensities of the reacted solutions were measured on an F-2500 fluorescence spectrophotometer with a polarizing filter. The excitation wavelength was 550 nm and the fluorescence intensity was recorded in different directions at an emission wavelength of 585 nm. The slit widths of the excitation and emission were 5 nm. The voltage was 700 V and the scanning speed was 3000 nm/min. All the samples were measured at room temperature. The *r* value was calculated using the following equations:

(1) $$r=\frac{I_{VV}-G\times I_{VH}}{I_{VV}+2G\times I_{VH}}$$

and

(2) $$G=\frac{I_{HV}}{I_{HH}}$$

where *I_VV_* is the vertical polarization excitation and vertical polarization emission, and *I_VH_* is the vertical polarization excitation and horizontal polarization emission. The instrumental correction factor *G* is the ratio of the sensitivity of the system to vertical polarization and horizontal polarization, which is related to the emission wavelength.

### 2.3. Polyacrylamide Gel Electrophoresis (PAGE)

All the DNA nanostructures were analyzed by 12% or 10% denaturing PAGE. Then, 10 μL of each DNA species (1 μmol/L) was mixed with 2 μL of loading buffer and loaded on the gel. All the gels were run at a constant voltage of 200 V for 30 min in 1× TBE buffer, followed by staining with 50 mL of 10% ethidium bromide for 15 min.

### 2.4. Synthesis of DNA Cross Scaffolds

The four DNA molecules (S1, S2, S3, and S4) required for synthesizing the DNA cross scaffold were dissolved in 1× PBS buffer solution. The concentration was measured with a Nanodrop 2000 spectrophotometer and the concentration of all the DNA solutions was adjusted to 10 μmol/L. The four DNA strands were mixed in an equimolar ratio in 1× PBS buffer to a final concentration for each DNA species of 1 μmol/L, and then the DNA solution was mixed thoroughly. Finally, the well-mixed solution was annealed by first denaturing at 95 °C for 5 min. This step used high-temperature denaturation to separate the DNA strands into a randomly coiled single stranded state, allowing the secondary structure to open. The solution was then cooled to 25 °C within 30 min. This step used rapid cooling to form the most stable complementary pairings of the single stranded DNA. Finally, the solution was incubated at 25 °C for 1 h to stabilize the newly formed DNA tetrahedral structure. The final annealed product was stored at 4 °C.

### 2.5. Steps for Testing HBV-DNA

First, hairpin 1 (H1) was annealed and stored in a refrigerator at 4 °C for later use. Then, 10 nmol/L Cas12a, 10 nmol/L crRNA, 50 nmol/L H1, 50 nmol/L DNA cross scaffolds, and HBV-DNA were mixed and incubated at 37 °C for 1 h. Next, the mixture was incubated at 70 °C for 20 min to inactivate Cas12a. Then, TDFs (50 nmol/L) were added, and the final volume was adjusted to 200 μL by adding 1× PBS buffer. After reacting for 30 min at room temperature, the fluorescence intensity of the solution was measured using an F-2500 fluorescence spectrophotometer with a polarizing filter and *r* was calculated.

### 2.6. Serum Sample Analysis

Human serum samples were acquired from Chongqing University Cancer Hospital, and their use strictly adhered to the ethics standards of Ethics Committee of Chongqing University Cancer Hospital (No. CZLS2023064-B). We obtained informed consent from blood donor volunteers of this study. HBV-DNA was added to the serum to obtain serum solutions with different concentrations of HBV-DNA. Finally, we used our method to detect HBV-DNA in the serum solutions, and the recovery rate was calculated.

## 3. Results

### 3.1. Principles of HBV-DNA Detection

**Figure 1:**
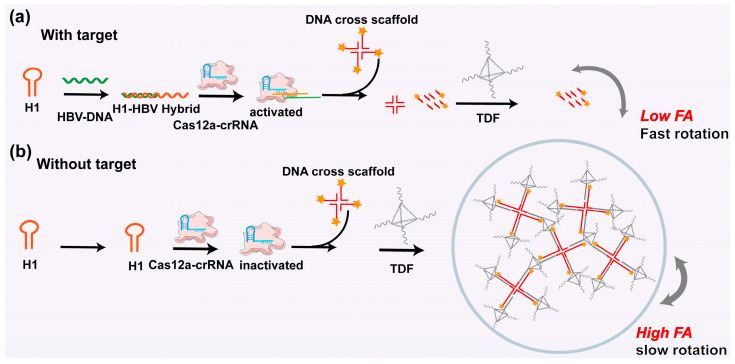
Illustration of the working principle of the FA method combining CRISPR/Cas12a with DNA tetrahedron amplification to detect HBV-DNA. (**a**) The FA response mechanism in the presence of HBV-DNA. (**b**) The FA response mechanism in the absence of HBV-DNA. H1-HBV hybrid represents the hybrid complex formed between hairpin DNA H1 and target HBV-DNA.

The detection principle is shown in Figure 1. First, TDF- and TAMRA-modified DNA cross scaffolds were pre-synthesized. When the target HBV-DNA was present, the hairpin H1 opened, and the exposed single-stranded portion of the scaffold activated CRISPR/Cas12a trans-cutting. The CRISPR system sheared the single-stranded portion of the DNA cross scaffold into small fragments with significantly smaller masses (or volume) and faster rotational speeds in the solution, resulting in a noticeably reduced *r* of TAMRA. In contrast, when the target HBV-DNA was absent, the CRISPR system was not activated because H1 did not open. At this time, the DNA cross scaffold remained intact and bound to the TDF as an assembly with a large mass (or volume) and slow rotation speed, and *r* of TAMRA was enhanced greatly. Therefore, sensitive detection of HBV-DNA was realized by a significant change in the *r* signal.

### 3.2. Characterization of DNA Nanostructures

**Figure 2:**
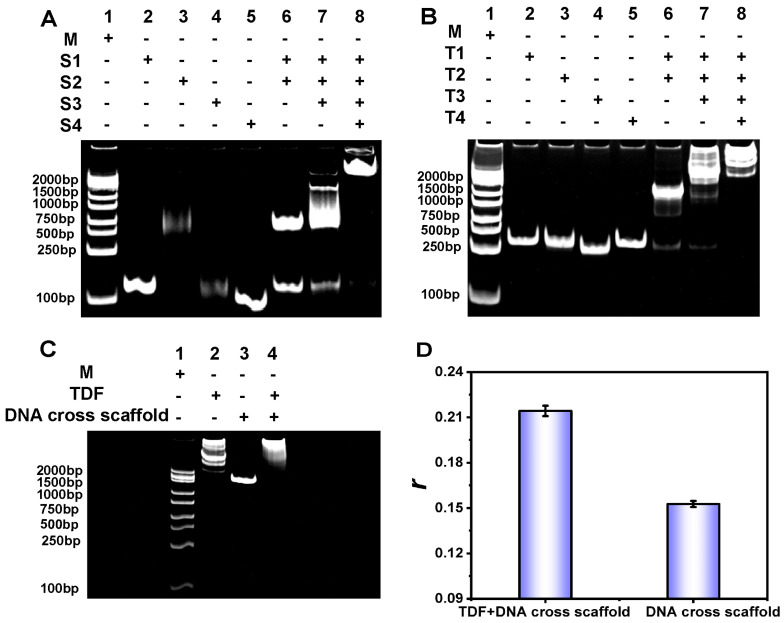
(**A**–**C**) PAGE analysis using a 12% gel. M is the marker. (**D**) Changes in *r* of the DNA cross scaffolds before and after adding TDFs. Each measurement was performed in triplicate (error bars indicate the standard deviation). Concentrations were as follows: HBV-DNA, 5 nmol/L; Cas12a, 10 nmol/L; crRNA, 10 nmol/L; TDFs, 50 nmol/L; and DNA cross scaffolds, 50 nmol/L.

We examined whether the DNA cross scaffolds and TDFs were successfully synthesized using 12% PAGE. As shown in Figure 2A, when all four ssDNAs (S1, S2, S3, and S4) that comprised the DNA cross scaffold were present, the migration rate of the corresponding band was the slowest, indicating that the DNA cross scaffold was successfully formed. Similarly, as shown in Figure 2B, when all four ssDNAs (T1, T2, T3, and T4) that comprised the TDFs were present, the corresponding band showed the slowest migration rate, which also confirmed the successful synthesis of the TDFs. The atomic force microscope (AFM) images further confirmed the successful formation of DNA cross scaffold and TDFs nanochain structures (Figure S1A,B).

We then used 10% PAGE to examine whether the TDF and the DNA cross scaffold assembled into a structure. As shown in Figure 2C, the reaction of TDFs with the DNA cross scaffolds produced band 4 with a slower migration rate than that of the other bands, confirming that TDFs and DNA cross scaffolds successfully complexed. The AFM images confirmed the successful formation of DNA cross scaffold and TDFs assembled nanochain structures (Figure S1C,D). We also measured the *r* of the DNA cross scaffolds after adding TDFs. As shown in Figure 2D, the *r* of the DNA cross scaffolds was significantly enhanced after adding TDFs, which indicated that the TDFs and DNA cross scaffolds successfully formed complexes, and demonstrated that the assembled complex was an ideal FA-enhanced material. We also investigated the chemical stability of the assembly of TDFs and DNA cross scaffolds. As shown in Figure S2, within the effective buffering range of PBS buffer, the change in pH did not cause a significant change in the signal value, indicating that the assembly structure of TDFs and DNA cross scaffolds has good chemical stability.

### 3.3. CRISPR/Cas12a Response

**Figure 3:**
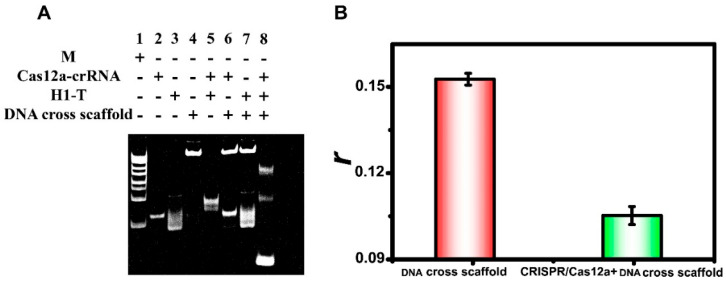
(**A**) PAGE analysis using a 12% gel. M is the marker and T is HBV-DNA. (**B**) Changes in *r* of the DNA cross scaffold before and after the addition of CRISPR/Cas12a. Each measurement was performed in triplicate (error bars indicate the standard deviation). Concentrations were as follows: HBV-DNA, 5 nmol/L; Cas12a, 10 nmol/L; crRNA, 10 nmol/L; TDFs, 50 nmol/L; DNA cross scaffolds, 50 nmol/L; and H1, 50 nmol/L.

To explore the shearing effect of the CRISPR/Cas12a system on the DNA cross scaffold, 12% PAGE analysis and the FA measurements were performed. As shown in Figure 3A, when the target HBV-DNA bound to Cas12a-crRNA, the DNA cross scaffold band disappeared and a new band with a faster migration rate was generated, indicating that HBV-DNA stimulated the trans-cutting activity of the CRISPR/Cas12a system. We also compared the changes in *r* of the DNA cross scaffold with the CRISPR/Cas12a system in its active and inactive states. As shown in Figure 3B, *r* was only significantly reduced in the presence of the activated Cas12a system, demonstrating that the reduction in the background signal was contingent upon Cas12a activation.

### 3.4. Feasibility Analysis

**Figure 4:**
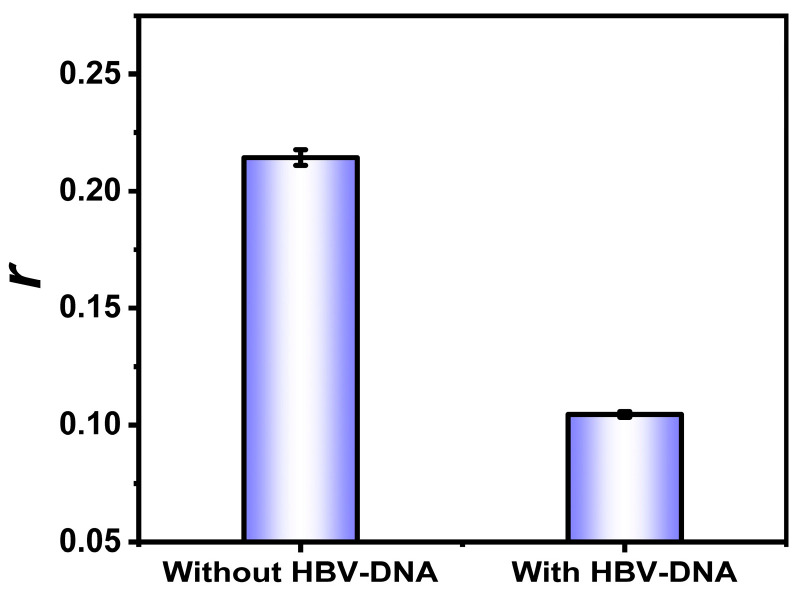
The feasibility analysis of the method. Each measurement was obtained in triplicate (error bars indicate the standard deviation). Concentrations were as follows: HBV-DNA, 5 nmol/L; Cas12a, 10 nmol/L; crRNA, 10 nmol/L; DNA cross scaffolds, 50 nmol/L; TDFs, 50 nmol/L; and H1, 50 nmol/L.

To investigate the feasibility of this method, we compared the response of *r* before and after adding HBV-DNA. As shown in Figure 4, *r* decreased significantly after adding HBV-DNA. This was because the addition of HBV-DNA activated CRISPR/Cas12a, which cut the DNA cross scaffolds. In contrast, in the absence of HBV-DNA, the complete structure of the DNA cross scaffold assembled into a larger structure with the TDF, producing a higher *r*. Therefore, this method detected HBV-DNA through a significantly decreased *r*.

### 3.5. Optimization of the Experimental Conditions

**Figure 5:**
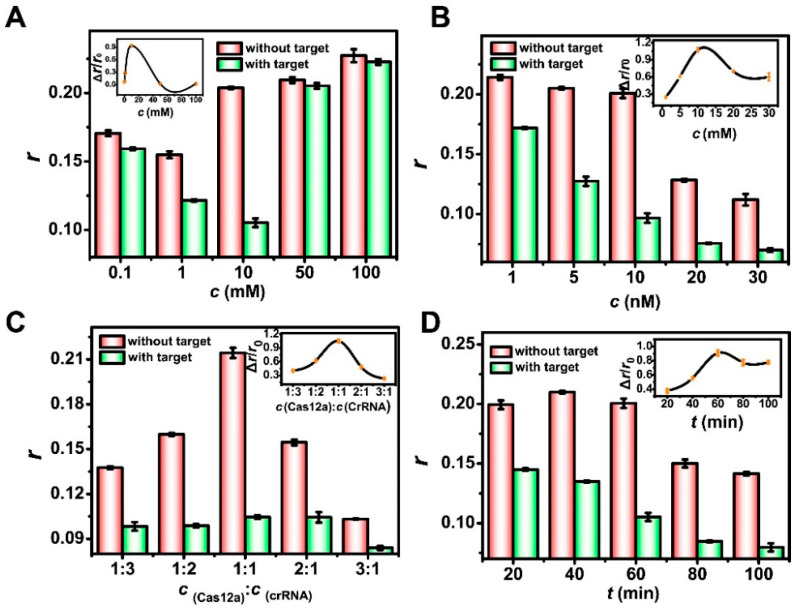
Optimization of the experimental conditions: (**A**) Mg^2+^ concentration; (**B**) Cas12a-crRNA complex concentration; (**C**) Cas12a to crRNA ratio; and (**D**) CRISPR/Cas12a system incubation time. Each measurement was obtained in triplicate (error bars indicate standard deviation). Concentrations were as follows: HBV-DNA, 5 nmol/L; Cas12a, 10 nmol/L; crRNA, 10 nmol/L; H1, 50 nmol/L; DNA cross scaffolds, 50 nmol/L; and TDFs, 50 nmol/L.

To optimize the experimental conditions, we first explored the effect of the Mg^2+^ concentration on the final signal. As shown in Figure 5A, Δ*r*/*r*_0_ (Δ*r* = *r*_0_ − *r*, where *r*_0_ and *r* are the values before and after the addition of HBV-DNA) first increased and then decreased as the concentration of Mg^2+^ increased. This was because when the concentration of Mg^2+^ was low, the DNA cross scaffold and TDF assembled incompletely; when the ion concentration was high, the shearing efficiency of the CRISPR system decreased, and the DNA cross scaffold was not cut, resulting in a low Δ*r*/*r*_0_.

Next, we investigated the effect of the concentration of the Cas12a-crRNA complex on the final signal. As shown in Figure 5B, Δ*r*/*r*_0_ reached a maximum value when the concentration of Cas12a-crRNA was 10 nmol/L. When the concentration of Cas12a-crRNA was low, the DNA cross scaffold was not completely cleaved, and thus *r* was not sufficiently reduced. However, when the concentration of Cas12a-crRNA was high, even if HBV-DNA was not added to activate the activity of Cas12a, the DNA cross scaffold was still cleaved to some degree, thus generating a background signal. Subsequently, we explored the effect of the concentration ratio of Cas12a to crRNA. As the concentration ratio of Cas12a to crRNA increased, Δ*r*/*r*_0_ tended to increase and then decrease. This suggested that when the ratio of crRNA was high, Cas12a caused cleavage activity even without the addition of the target, which eventually produced a higher background signal. As shown in Figure 5C, the optimal ratio of Cas12a-crRNA was 1:1. In addition, the reaction time of the CRISPR/Cas12a system was also an important influencing factor. When the reaction time was short, the DNA cross scaffold was not completely sheared, while a reaction time that was long easily produced a high background signal. As shown in Figure 5D, Δ*r*/*r*_0_ reached its maximum value when the reaction time of the CRISPR/Cas12a system was 30 min.

### 3.6. Sensitivity and Selectivity of HBV-DNA Detection

**Figure 6:**
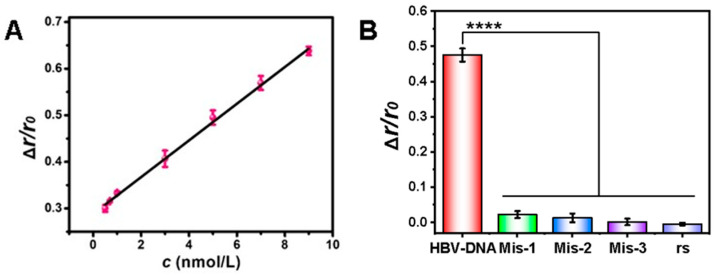
(**A**) Linear relationship between Δ*r*/*r*_0_ and the concentration of HBV-DNA. (**B**) Selectivity of the method. Concentrations were as follows: Cas12a, 10 nmol/L; crRNA, 10 nmol/L; H1, 50 nmol/L; DNA cross scaffolds, 50 nmol/L; and TDFs, 50 nmol/L. The asterisk indicates significant differences with **** *p* < 0.0001.

After investigating the optimal experimental conditions, we explored the Δ*r*/*r*_0_ signaling response using different concentrations of HBV-DNA. As shown in Figure 6A, Δ*r*/*r*_0_ was positively correlated with the HBV-DNA concentration in the range of 0.5–9 nmol/L with a linear regression equation of Δ*r*/*r*_0_ = 0.039*c* + 0.29 (where c is the HBV-DNA concentration, nmol/L) and correlation coefficient (R^2^) of 0.997. The LOD was calculated to be 48 pmol/L (3*σ*/*k*), which was much lower than that of several traditional detection methods (Table 1).

Next, we examined the selectivity of this method. Under the same experimental conditions, we investigated five different DNA species, including HBV-DNA, mis-1, mis-2, mis-3, and a random sequence (rs). As shown in Figure 6B. the highest Δ*r*/*r*_0_ values were obtained only when HBV-DNA was added, and the Δ*r*/*r*_0_ signals of all the mismatched base sequences were much lower than that of HBV-DNA. Even with only a single base mismatch, the observed Δ*r*/*r*_0_ value remained low. This suggested that theoretically, HBV-DNA from different serotypes or isolates may also exhibit reduced Δ*r*/*r*_0_ values as a result of variations in their DNA sequences, indicating that the method had good selectivity.

Apart from the remarkable sensitivity and selectivity showcased in this article, this work also boasts several inherent advantages over other detection methods. Firstly, in contrast to electrochemical-based detection, which typically necessitates intricate electrode modifications and non-specific adsorption, our FA-based uniform detection is conducted in solution, thereby obviating the need for cumbersome washing procedures and surface engineering. Secondly, the FA signal is predicated on the rotational speed of molecules, rendering it inherently less vulnerable to fluctuations in light source intensity or environmental background fluorescence compared to intensity-based fluorescence detection. This confers significant benefits in terms of the measurement’s robustness and reproducibility.

### 3.7. Interference During HBV-DNA Detection

**Figure 7:**
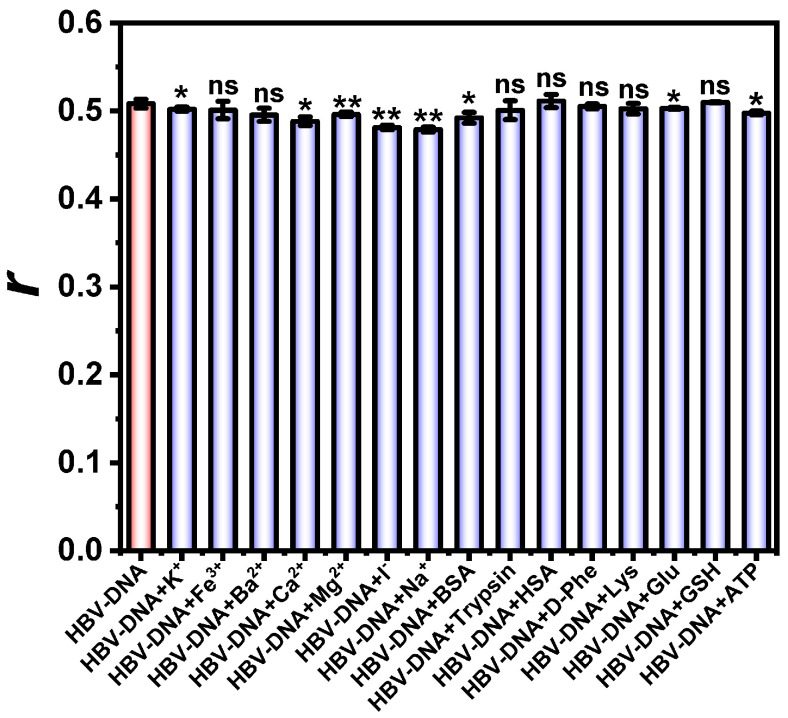
Susceptibility of the method to interference. Concentrations: Cas12a, 10 nmol/L; crRNA, 10 nmol/L; H1, 50 nmol/L; DNA cross scaffolds, 50 nmol/L; TDFs, 50 nmol/L; and K^+^, Fe^3+^, Ba^2+^, Ca^2+^, Mg^2+^, I^−^, Na^+^, BSA, trypsin, HSA, D-Phe, Lys, Glu, GSH, and ATP, 50 nmol/L. The asterisk indicates significant differences with * *p* < 0.05, ** *p* < 0.01, and *** *p* < 0.001; ns indicates no significant difference.

We also explored the anti-interference ability of this method. We added various types of interfering substances, including metal ions, amino acids, and proteins, to the HBV-DNA solution, with a ratio of the concentration of the interfering substances to the target of 10:1. As shown in Figure 7, the addition of the interfering substances did not cause any significant change in the signal value, indicating that our proposed method was not very susceptible to interference.

### 3.8. Detection of HBV-DNA in Human Serum

To investigate the accuracy of the method in real samples, we conducted a spiked recovery experiment. First, different concentrations of HBV-DNA were added to serum samples, and the proposed method was used to determine the concentration of HBV-DNA in the serum samples. Finally, the effectiveness of the method on real samples was evaluated by calculating the recovery rate. As shown in Table 2, we found that the signal was enhanced as the concentration of HBV-DNA in human serum increased. Moreover, the calculated recoveries for its detection ranged from 97.6% to 104.1% (*n* = 3), indicating the good performance of our method. Therefore, this method has the potential to be applied to clinical blood samples for HBV-DNA detection.

## 4. Conclusions

In summary, a novel FA amplification method was developed for the highly sensitive detection of HBV-DNA by leveraging the synergistic effects of CRISPR-Cas12a and a DNA tetrahedron assembly. The CRISPR-Cas12a system provided exceptional specificity and significantly reduced the background signal through its efficient collateral cleavage activity, while the DNA tetrahedron nanostructure served as an ideal FA signal enhancer due to its precisely controlled architecture and high loading capacity of fluorophores. The integration of these two elements resulted in a remarkable improvement in the SBR, allowing for the sensitive detection of HBV-DNA. Notably, this method demonstrated outstanding sensitivity in an FA-based biosensing application. The established platform successfully detected HBV-DNA in complex samples with high sensitivity and accuracy, showing great potential for clinical diagnostic applications.

## Figures and Tables

**Table 1:** Comparison of the proposed method with other methods.

Method	Linear Range	LOD	References
Electrochemistry	10 nmol/L–500 nmol/L	1 nmol/L	[38]
Electrochemistry	0.1 nmol/L–1 μmol/L	0.1 nmol/L	[39]
Fluorescence	4 nmol/L–625 nmol/L	0.97 nmol/L	[40]
Fluorescence	0 nmol/L–500 nmol/L	4 nmol/L	[41]
FA	0.5 nmol/L–9 nmol/L	48 pmol/L	This work

**Table 2:** Recovery experiment analyzing HBV-DNA determination in human serum.

Sample	Add (nmol/L)	Found (nmol/L)	Recovery (%)	RSD (%)
Human serum	2	2.03 ± 0.08	97.6–104.1	4.8
	5	5.05 ± 0.11	99.6–103.3	6.1
	8	8.10 ± 0.15	98.9–103.2	9.9

## Data Availability

The data that support the findings of this study are available upon request.

## Supplementary Materials

The following supporting information can be downloaded at: https://www.mdpi.com/article/10.3390/bios15100700/s1, Table S1: Nucleic Acid Sequence; Figure S1: AFM images; Figure S2: Stability of DNA cross scaffold and TDFs assembly.

## Abbreviations

The following abbreviations are used in this manuscript:

FA	fluorescence anisotropy
FP	fluorescence polarization
ssDNA	single-stranded deoxyribonucleic acid
RNA	ribonucleic acid
CRISPR	clusters of regularly interspaced short palindromic repeats
Cas	correlated protein
SBR	signal-to-background ratio
TDF	tetrahedral DNA frameworks
HBV-DNA	hepatitis B virus DNA
LOD	limit of detection
DEPC	diethyl pyrocarbonate
PBS	phosphate-buffered saline
TBE	tris-borate ethylene diamine tetraacetic acid
RSD	relative standard deviation
D-Phe	D-phenylalanine
Glu	glutamic acid
GSH	glutathione
Lys	lysine
ATP	adenosinetriphosphate
BSA	bovine serum albumin
HSA	human serum albumin
PAGE	polyacrylamide gel electrophoresis

## References

[1] Huang Q., Chen B., Shen J., Liu L., Li J., Shi J., Li Q., Zuo X., Wang L., Fan C. (2021). Encoding fluorescence anisotropic barcodes with DNA frameworks. J. Am. Chem. Soc..

[2] Lakowicz J.R., Masters B.R. (2008). Principles of fluorescence spectroscopy, Third Edition. J. Biomed. Opt..

[3] Chen J., Liu J., Chen X., Qiu H. (2019). Recent progress in nanomaterial-enhanced fluorescence polarization/anisotropy sensors. Chin. Chem. Lett..

[4] Fan Y.L., Liu Z.Y., Zeng Y.M., Huang L.Y., Li Z., Zhang Z.L., Pang D.W., Tian Z.Q. (2021). A near-infrared-II fluorescence anisotropy strategy for separation-free detection of adenosine triphosphate in complex media. Talanta.

[5] Zhang D., Fu R., Zhao Q., Rong H., Wang H. (2015). nanoparticles-free fluorescence anisotropy amplification assay for detection of RNA nucleotide-cleaving DNAzyme activity. Anal. Chem..

[6] Zhang X., Xu J., Xing X., Yao L., Shang H., Chen W. (2021). Framework nucleic acid-wrapped protein-inorganic hybrid nanoflowers with three-stage amplified fluorescence polarization for terminal deoxynucleotidyl transferase activity biosensing. Biosens. Bioelectron..

[7] Fan Y.Y., Mou Z.L., Wang M., Li J., Zhang J., Dang F.Q., Zhang Z.Q. (2018). Chimeric aptamers-based and MoS_2_ nanosheet-enhanced label-free fluorescence polarization strategy for adenosine triphosphate detection. Anal. Chem..

[8] Yang B., Zhang X.B., Kang L.P., Shen G.L., Yu R.Q., Tan W. (2013). Target-triggered cyclic assembly of DNA–protein hybrid nanowires for dual-amplified fluorescence anisotropy assay of small molecules. Anal. Chem..

[9] Ma P., Guo H., Duan N., Ma X., Yue L., Gu Q., Wang Z. (2021). Label free structure-switching fluorescence polarization detection of chloramphenicol with truncated aptamer. Talanta.

[10] Zhu Q., Li H., Xu D. (2020). Sensitive and enzyme-free fluorescence polarization detection for miRNA-21 based on decahedral sliver nanoparticles and strand displacement reaction. RSC Adv..

[11] Chullipalliyalil K., Elkassas K., McAuliffe M.A.P., Vucen S., Crean A. (2023). In-vial detection of protein denaturation using intrinsic fluorescence anisotropy. Anal. Chem..

[12] Yakovchuk P. (2006). Base-stacking and base-pairing contributions into thermal stability of the DNA double helix. Nucleic Acids Res..

[13] Hermann T., Patel D.J. (2000). Adaptive recognition by nucleic acid aptamers. Science.

[14] Pabo C. (1984). Protein-DNA recognition. Ann. Rev. Biochem..

[15] Schweizer K., Léon J.C., Ravoo B.J., Müller J. (2016). Thermodynamics of the formation of Ag(I)-mediated azole base pairs in DNA duplexes. J. Inorg. Biochem..

[16] Ma X., Zhang Y., Qiao X., Yuan Y., Sheng Q., Yue T. (2023). Target-induced AIE Effect coupled with CRISPR/Cas12a system dual-signal biosensing for the ultrasensitive detection of gliotoxin. Anal. Chem..

[17] Liang M., Li Z., Wang W., Liu J., Liu L., Zhu G., Karthik L., Wang M., Wang K.F., Wang Z. (2019). A CRISPR-Cas12a-derived biosensing platform for the highly sensitive detection of diverse small molecules. Nat. Commun..

[18] Lau C.H., Liang Q.L., Chen X., Li J., Shi Y., Huang Y., Xia Q., Zhu H., Chen G. (2025). CRISPR/Dx-Based Colorimetric and Electrochemical Detection Systems for POCT Applications. Biosens. Bioelectron..

[19] Zhang Z.X., Rong X.X., Xie T.J., Li Z.H., Song H.Z., Zhen S.J., Wang H.F., Wu J.H., Jaffrey S.R., Li X. (2024). Fluorogenic CRISPR for Genomic DNA Imaging. Nat. Commun..

[20] Zhou Y., Che S., Wang Z., Zhang X., Yuan X. (2024). Primer Exchange Reaction Assisted CRISPR/Cas9 Cleavage for Detection of Dual microRNAs with Electrochemistry Method. Microchim. Acta.

[21] Wei L., Luo S., Zhou W., Ren B., Li M., Liang L., Li X., Wei G. (2025). Rapid Detection of Pseudomonas Aeruginosa by Glycerol One-Pot RAA/CRISPR-Cas12a Method. Front. Chem..

[22] Cao M., Zhang X., Liu S., Wang S., Li X., Zhang X., Li B., Huang H. (2025). Signal Amplified Fluorometric Detection of Anti-CRISPR Proteins Based on Rolling Circle Amplification. Sens. Actuators B.

[23] Nie Y., Li X., Yang W., Fei S., Wang Y., Li Y., Zhang K., Kang J., Cheng Y., Wang H. (2025). Concanavalin-A-Assisted Extraction-Free One-Pot RPA–CRISPR/Cas12a Assay for Rapid Detection of HPV16. Microchim. Acta.

[24] Liu G. (2025). Advancing CRISPR/Cas Biosensing with Integrated Devices. ACS Sens..

[25] Li Z., Su X., Lin Y., Zhang Y., Zhang A., Wu X., Jiyu X., Li Q., Wei Z. (2025). Expanding the Cell Quantity of CRISPR/Cas9 Gene Editing by Continuous Microfluidic Electroporation Chip. Bioelectrochemistry.

[26] Zhuang S., Dong Y., Huang X., Liu T., Gu D., Liu Y. (2025). A Universal Cas12a Activity Regulation Strategy via Photocleavable crRNA 3′-Terminal Additives Enables One-Pot CRISPR Detection of Arboviruses. Chem. Eng. J..

[27] Dong J., Qi L., Wu X., Sun C., Hu Q., Su Y., Shao G., Zhang Y., Meng F., Du Y. (2024). An Electrochemical, Fluorescent, and Colorimetric Triple-Mode Homogeneous Biosensor for the Simultaneous Detection of Influenza A, Influenza B, and SARS-CoV-2. Sens. Actuators B.

[28] Zheng X., Yao S., Yin C., Zhao H., Wang J., Su T., Li H., Wang J., Zhao C. (2025). CRISPR-Integrated Nanoconfined Interparticle Catalytic Hairpin Assembly for Enhanced Dual-Mode SARS-CoV-2 Detection in Wastewater. Biosens. Bioelectron..

[29] De J., Mukama O., Amissah O.B., Sun Y., Karangwa E., Liu Y., Mugisha S., Cheng N., Wang L., Chen J. (2023). A rationally designed CRISPR/Cas12a assay using a multimodal reporter for various readouts. Anal. Chem..

[30] Yi Z., De J., Mukama O., Li Z., Odiwuor N., Jing H., Nie C., Hu M., Lin Z., Wei H. (2021). Rational programming of Cas12a for early-stage detection of COVID-19 by lateral flow assay and portable real-time fluorescence readout facilities. Biosensors.

[31] Habimana J.D.D., Huang R., Muhoza B., Kalisa Y.N., Han X., Deng W., Li Z. (2022). Mechanistic insights of CRISPR/Cas nucleases for programmable targeting and early-stage diagnosis: A review. Biosens. Bioelectron..

[32] Wu C., Chen Z., Li C., Hao Y., Tang Y., Yuan Y., Chai L., Fan T., Yu J., Ma X. (2022). CRISPR-Cas12a-empowered electrochemical biosensor for rapid and ultrasensitive detection of SARS-CoV-2 delta variant. Nano Micro Lett..

[33] Kellner M.J., Koob J.G., Gootenberg J.S., Abudayyeh O.O., Zhang F. (2019). Sherlock: Nucleic acid detection with CRISPR nucleases. Nat. Protoc..

[34] Shen J., Chen Z., Xie R., Li J., Liu C., He Y., Ma X., Yang H., Xie Z. (2023). CRISPR/Cas12a-assisted isothermal amplification for rapid and specific diagnosis of respiratory virus on an microfluidic platform. Biosens. Bioelectron..

[35] Duan M., Li B., He Y., Zhao Y., Liu Y., Zou B., Liu Y., Chen J., Dai R., Li X. (2024). A CG@MXene nanocomposite-driven e-CRISPR biosensor for the rapid and sensitive detection of salmonella typhimurium in food. Talanta.

[36] Xie T.J., Xie J.L., Luo Y.J., Mao K., Huang C.Z., Li Y.F., Zhen S.J. (2023). CRISPR-Cas12a coupled with DNA nanosheet-amplified fluorescence anisotropy for sensitive detection of biomolecules. Anal. Chem..

[37] Xie J.L., Xie T.J., Luo Y.J., Mao K., Huang C.Z., Li Y.F., Zhen S.J. (2024). Octopus-like DNA nanostructure coupled with graphene oxide enhanced fluorescence anisotropy for hepatitis B virus DNA detection. Chin. Chem. Lett..

[38] Xiang Q., Huang J., Huang H., Mao W., Ye Z. (2018). A label-free electrochemical platform for the highly sensitive detection of hepatitis B virus DNA using graphene quantum dots. RSC Adv..

[39] Zhu H.T., Wang J.X., Xu G.Y. (2009). Fast synthesis of Cu_2_O hollow microspheres and their application in DNA biosensor of hepatitis B virus. Cryst. Growth Des..

[40] Liu Z.C., Zhang L., Zhang Y.M., Liang R.P., Qiu J.D. (2014). Exonuclease III-assisted recycling amplification detection of hepatitis B virus DNA by DNA-scaffolded silver nanoclusters probe. Sens. Actuators B.

[41] Wang X., Lou Y., Wang Q., Guo Z., Fang X., Zhong H., Mao Q., Jin L., Wu H., Zhao Q. (2010). Ds-DNA nanosensor for the detection of hepatitis B virus DNA and the single-base mutants. Biosens. Bioelectron..

