# Supplementary material for: CRISPR-Cas12a and DNA Tetrahedron Assemblies Amplified Fluorescence Anisotropy for the Sensitive Detection of Hepatitis B Virus DNA
Source: Biosensors (Basel). 2025 Oct 17;15(10):700. doi: 10.3390/bios15100700 (PMC12563765; doi:10.3390/bios15100700)
Supplement: Supplementary file 1 [file biosensors-15-00700-s001.zip › biosensors-3876136-supplementary.pdf]

Electronic Supporting Information

CRISPR-Cas12a and DNA Tetrahedron Assemblies Amplified Fluorescence Anisotropy for the Sensitive Detection of Hepatitis B Virus DNA

Yu Qin [1,2,3], Jiali Xie [1,2,3] and Shujun Zhen [1,2,3,*]

1 College of Chemistry and Chemical Engineering, Southwest University, Chongqing 400715, China; swuqy1@email.swu.edu.cn (Y.Q.); xji0208@email.swu.edu.cn (J.X.)

2 Key Laboratory of Biomedical Analytics of Chongqing Science and Technology Bureau, Southwest University, Chongqing 400715, China

3 Academy for Advanced Interdisciplinary Studies, Southwest University, Chongqing 400715, China

* Correspondence: zsj@swu.edu.cn

1. Oligonucleotide sequences

Table S1. Nucleic Acid Sequence.

Name	Sequence (5' to 3')
H1	TTC AGT TAT ATG GAT CGT ACG TAA TCC ATA TAA CTG AAA GCC AA
HBV-DNA	TTG GCT TTC AGT TAT ATG GAT GAT GTG GTA
crRNA	UAA UUU CUA CUA AGU GUA GAU ACG UAC GAU CCA UAU AAC U
S1	AGG GTA TCC CTA AGC TCC ATC GAT GTA GTT TCG GTT CCT A-TAMRA
S2	GCT ACA TCG ATG GAG CCG CTA GGC CTA GTT TCG GTT CCT A-TAMRA
S3	CTA GGC CTA GCG TCC GGA ATT CTT TGT TTC GGT TCC TA-TAMRA
S4	TCA AAG AAT TCC GGA TTA GGG ATA CCC TTT CGG TTC CTA-TAMRA
T1	TTT CGG TTC CTA ACA TTC CTA AGT CTG AAA CAT TAC AGC TTG CTA CAC GAG AAG AGC CGC CAT AGT A
T2	TTT CGG TTC CTA TAT CAC CAG GCA GTT GAC AGT GTA GCA AGC TGT AAT AGA TGC GAG GGT CCA ATA C
T3	TTT CGG TTC CTA TCA ACT GCC TGG TGA TAA AAC GAC ACT ACG TGG GAA TCT ACT ATG GCG GCT CTT C
T4	TTT CGG TTC CTA TTC AGA CTT AGG AAT GTG CTT CCC ACG TAG TGT CGT TTG TAT TGG ACC CTC GCA T
Mis-1	TTG CCT TTC AGT TAT ATG GAT GAT GTG GTA
Mis-2	TTG CCT TTC ACT TAT ATG GAT GAT GTG GTA
Mis-3	TTG CCT TTC ACT TAT ATG GAT GAT CTG GTA
HIV-DNA	ATA CCA CAT CAT CCA TAT AAC TGA AAG CCA

2. AFM Characterization and stability

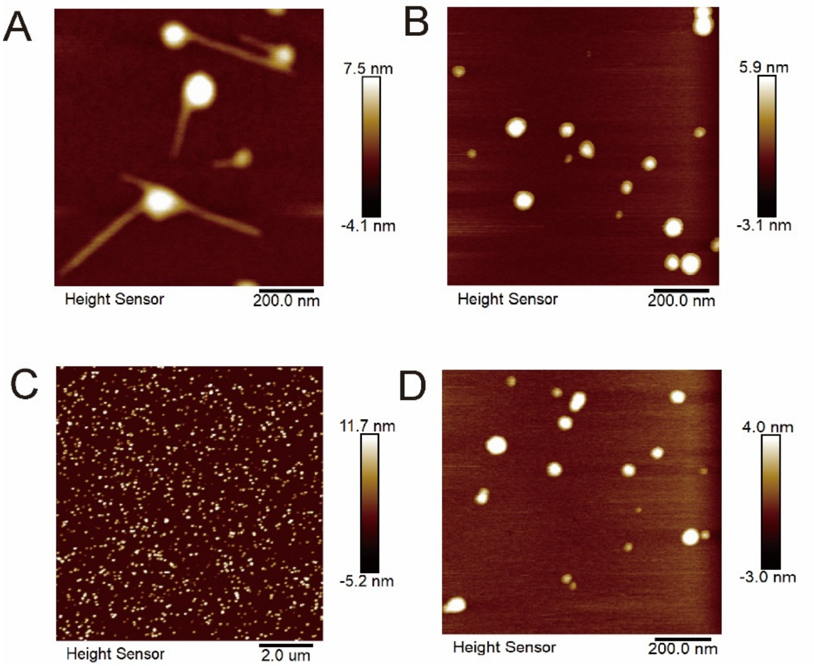

Figure S1. AFM images of TDFs (A), DNA cross scaffold (B), and the assembly structure of DNA cross scaffold and TDFs (C-D).

3. Stability of DNA cross scaffold and TDFs assembly

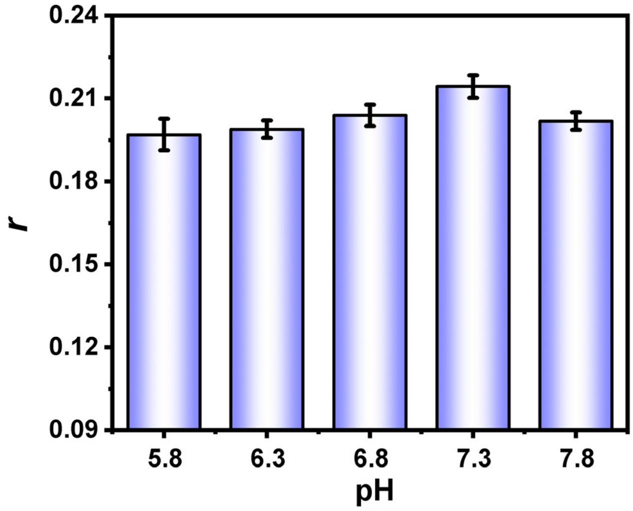

Figure S2. Stability of DNA cross scaffold and TDFs assembly. Each measurement was performed in triplicate (error bars indicate the standard deviation). Concentrations: TDFs, 50 nmol/L; and DNA cross scaffolds, 50 nmol/L.
